# Outdoor Air Pollution and Gestational Diabetes Mellitus: A Systematic Review and Meta-Analysis

**Published:** 2019-01

**Authors:** Mohamed H. ELSHAHIDI

**Affiliations:** Faculty of Medicine, Mansoura University, Mansoura, Dakahliya, Egypt

**Keywords:** Gestational diabetes, Air pollution, Pregnancy, Complications, Prevention

## Abstract

**Background:**

During the past 20 years, the prevalence of gestational diabetes mellitus (GDM) has increased by ∼10%–100% in several race/ethnicity groups. There is an association between ambient air pollution (AAP) and GDM. This study aimed to **s**ummarize the evidence about the association between AAP and GDM.

**Methods:**

PubMed, Embase, Scopus, Web of Science and Cochrane Library were searched from inception till Oct 2017. Studies about the association between ambient air pollutants levels and GDM were included. Pooled effect estimates and their 95% confidence interval (CI) were calculated using *R*.

**Results:**

Eight studies met the inclusion criteria. The odds of developing GDM upon exposure to CO (per 1 ppm), NO (per 1 ppb), NO2 (per 10 μg/m3), NOx (per 1 ppb), O3 (per 10 ppb), SO2 (per 10 ppb), PM10 (per 10 μg/m3) and PM2.5 (per 10 μg/m3) were 1.47 (95% CI 0.88–2.06), 1.04 (95% CI 1.03–1.06), 1 (95% CI 0.93–1.08), 1.02 (95% CI 1–1.04), 1.05 (95% CI 0.94–1.16), 1.39 (95% CI 1.04–1.73), 0.97 (95% CI 0.94–0.99) and 1.12 (95% CI 0.93–1.31), respectively.

**Conclusion:**

The current literature showed evidence for an association between AAP and GDM. However, further well-designed studies are needed.

## Introduction

Pregnancy is a vulnerable period for women because of the increased insulin resistance by the placenta diabetogenic effects in order to ensure more available glucose to the fetus (1). Gestational diabetes mellitus (GDM) is a condition defined as any degree of glucose intolerance during pregnancy that resolves postpartum (2). GDM complicates nearly more than 10% of all pregnancies in the USA and 17% of pregnancies in Iran (3). During the past 20 years, the prevalence of GDM has increased by ∼10%–100% in several race/ethnicity groups (4). The rise in GDM along with type 2 diabetes (T2D) and obesity worldwide has become of particular concern (5). In different populations and geographical regions, the risk of developing T2D is 7.4 higher among women with GDM in comparison with women without GDM, both postpartum and later in life (6–8). In addition, young women with GDM are at greater risk of developing cardiovascular diseases (CVD) and coronary artery diseases (CAD), much attributable to the development of T2D (9,10). Moreover, there is reportedly increased risk of preeclampsia, asymptomatic bacteriuria, pyelonephritis and cesarean delivery among women with GDM (11,12). Besides the adverse effects of GDM on the mother both in the short and long-terms, GDM was linked with many fetal and neonatal complications including macrosomia (1–16), shoulder dystocia (17), neonatal hypoglycemia (18) and congenital malformation (19–21).

With 92% of the global population living in areas not meeting the WHO air quality guidelines levels, ambient (outdoor) air pollution (AAP) is being considered a major risk to the public health (22). In 2012, AAP caused 3 million premature death worldwide, with 88% of these premature deaths in low and middle-income (LMI) countries (22). AAP was associated with many adverse health conditions including cardiopulmonary disease, lung cancer and acute lower respiratory infection (23,24). In addition, air pollution was significantly associated with insulin resistance and diabetes-related mortality (25–27). This association may be gender-dependent, being more distinct among women than men. (28–31). Although the biological mechanisms underlying this association are still unclear, animal studies have shown that high levels of air pollution may be equivalent to a high-fat diet in terms of its effects, involving immune activation, endoplasmic reticulum (ER) stress, oxidative stress and CNS inflammation (32,33).

Some studies have investigated the association between GDM and air pollutant including nitric oxides (NO_x_), sulfur dioxide (SO_2_), ozone (O_3_), particulate matter with diameter ≤ 10μm (PM_10_) and particulate matter with diameter ≤ 2.5 (PM_2.5_). However, the evidence remains controversial and inconsistent (34).

This systematic review and meta-analysis aimed to summarize the current evidence regarding the association between ambient air pollution and gestational diabetes mellitus. In addition, it will help to provide further rationale for improving air quality standard and the public health.

## Methods

### Guidelines

The Meta-analysis of observational studies in epidemiology (MOOSE) statement was used in reporting this meta-analysis (35).

### Search Strategy

The search process was carried out using the following keywords: (gestational diabetes OR pregnancy induced diabetes* OR pregnancy diabetes* OR gestational diabetes* OR GDM) and (air pollution OR outdoor air pollution OR ambient air pollution OR traffic pollution OR air pollutants OR outdoor air pollutants OR nitrogen dioxide OR particulate matter OR sulfur dioxide OR ozone). No language nor publication type filters were used.

### Data sources

An electronic search on PubMed, Embase (via ovidSP), Web of Science, Scopus and Cochrane library databases was conducted from their dates of inception till Oct 2, 2017. Moreover, references of included articles were handsearched for relevant records.

### Criteria for selecting studies

Retrieved records were screened in two steps: title and abstract screening then full-text reviewing. Records were screened for meeting the inclusion criteria: 1) peer-reviewed, published article 2) human population 3) observational study providing data about the association between GDM and AAP 4) levels of air pollutants were monitored. Exclusion criteria were: 1) conference abstracts, editorial, commentaries or reviews 2) in vitro or animal study 3) not measuring levels of air pollutants or use proximity to roads as an index for air quality 4) indoor air pollution study.

### Data extraction

A data extraction form was prepared to collect the following data: study ID (first author name, year of publication), country, duration of the study, data source of the study, sample size, number of GDM cases, date of diagnosing GDM, exposure measurement method, investigated pollutants and data type.

### Quality assessment

A modified version of the Newcastle-Ottawa Scale (NOS) for assessing the quality of non-randomized studies in meta-analyses was used to assess the quality of the included studies (36). Each study could attain a maximum of four points. Studies with ≥ 3 points were considered of good quality. Those with ≤ 2 points were considered of poor quality. The scale rated the papers according to 1) sample representativeness 2) use of reliable GDM diagnostic method 3) residential-level air quality monitoring 4) adjustment for potential covariates; maternal age and BMI.

### Data analysis

Effect estimates of the included studies were pooled and meta-analyzed using the ‘*meta*’ package, *R* (version 3.4.0) (37). Because most studies used odds ratio (OR) as their effect estimate, the pooled effect estimates were reported as OR with their 95% confidence interval (CI). Heterogeneity among included studies was tested by the Cochran-Q test and quantified its extent by the I-square test. When a significant heterogeneity (*P*<0.1) was found, the pooled effect estimate was calculated under the random-effect model (38). Since included studies reported effect estimate (EE) with different pollutant increments, commonly used standardized increments (10 part per billion (ppb) in O_3_, 10 μg/m^3^ in PM_10_, 10 μg/m^3^ in PM_2.5_, 1 part per million (ppm) in CO, 1 ppb in NO, 10 μg/m^3^ in NO_2_, 1 ppb in NO_x_ and 10 ppb in SO_2_) were calculated for each study using the following equation: ${\text{EE}}_{\left(\text{standardized}\right)}={{\text{EE}}_{\left(\text{original}\right)}}^{\text{increment}(10)/\text{increment(original)}}$ (27, 39, 40). Because the number of the included studies was <10, publication bias assessment was not reliable according to Egger and his colleagues (41).

## Results

### Study selection

**Fig.1:**
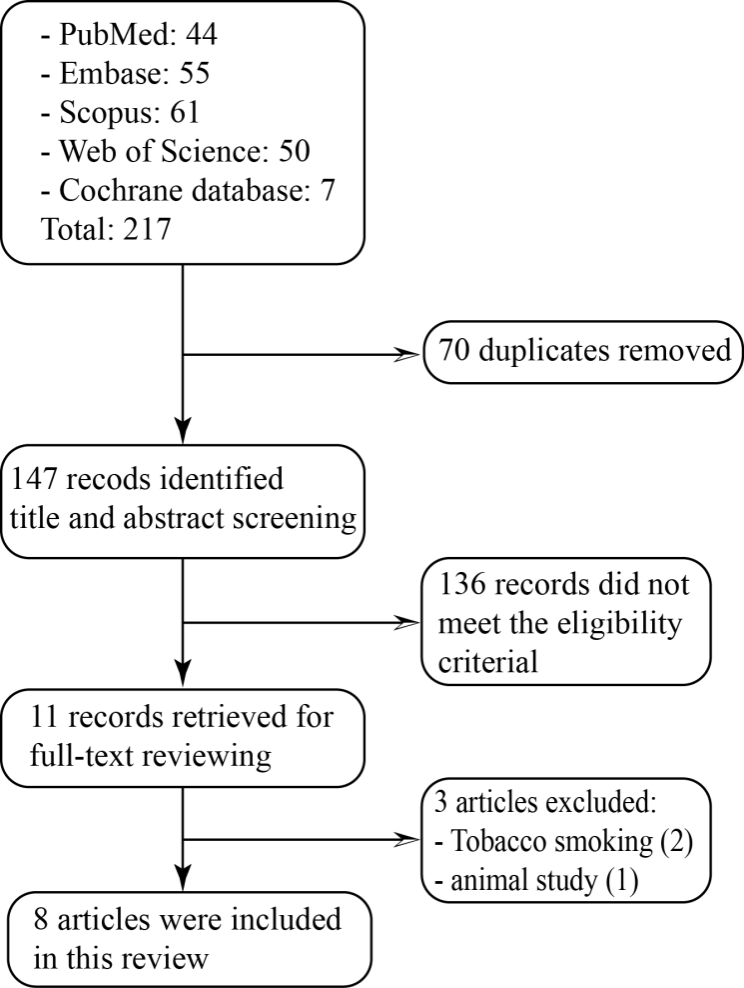
Flow diagram of the search process

The search strategy retrieved 247 references. After abstract and full-text reviewing, 8 articles met our inclusion criteria (Fig. 1).

A summary of the included studies was presented in Table 1 (42–49).

**Table 1:** Characteristics of the included studies

***No.***	***Study ID***	***Country***	***Duration***	***Data sources***	***Study Size***	***GDM cases***	***Date of GDM diagnosis*** ^*^	***Exposure measurement***	***Pollutants*** ^**^	***Data type*** ^***^	***Quality score***
1	Pan et al., 2017 ( 42 )	Taiwan	2005	Birth Registration Database	19606	378	Week 17 and beyond	Resident level estimation using ArcGIS model	PM_10_, CO, NO, NO_2_, NO_x_, SO_2_, O_3_	10 μg/m^3^ increments in PM_10_, 0.1 ppm in CO, 1 ppb in NO, NO_2_, NOx, SO_2_ and O_3_	4
2	M. Pedersen et al., 2017 ( 43 )	Denmark	1996–2002	Danish National Birth Cohort	72745	565	Week 6–10	Pollutant was measured using address-level AirGIS dispersion model	NO_2_	Per 10 μg/m^3^ NO_2_	4
3	Fleisch et al., 2014 ( 44 )	USA	1999–2002	All Boston-area women at their first prenatal visit to Harvard Vanguard Medical Associates were invited	2093	118	Second trimester	Residential-level model	PM_2.5_ and Black carbon	IQR	4
4	Hu et al., 2015 ( 45 )	USA	2004–2005	Bureau of Vital Statistics and Office of Health Statistics and Assessment, Florida Department of Health	410267	14032	Week 24–28	Residential-level model using ArcGIS model	PM_2.5_ and O_3_	5-μg/m^3^ increase in PM_2.5_, 5 ppb increase in exposure to O_3_	4
5	Malmqvist et al., 2013 ( 46 )	Sweden	1999–2005	Swedish Medical Birth Registry	81110	1599	During week 24, and at week 10 if previous family history of diabetes or previous diagnosis of gestational diabetes	AERMOD model gathering data from line, point and area sources linked to woman address	NO_x_	Per quartiles	3
6	Robledo et al., 2015 ( 47 )	USA	2002–2008	Consortium on Safe Labor (CSL) cohort study	219952	11334	Week 24 – 28	Maternal exposures are based on the average air pollutant levels for her delivery hospital referral region	PM_2.5_, CO, NO_x_, SO_2_, O_3_	IQR	3
7	Fleisch et al., 2016 ( 48 )	USA	2003–2008	Massachusetts Registry of Vital Records and Statistics	159373	5381	Week 24 – 28	Residential-level, satellite-based spatiotemporal model	PM_2.5_	10–90 percentile range	4
8	Yu-Ting Lin et al., 2014 ( 49 )	Taiwan	2001–2007	Taiwanese Birth Registry	86224	2198	Week 24 – 28	Air quality monitoring stations using ArcGIS model	O_3_	10 ppb for ozone (O_3_)	3

* Week of gestation

** PM _10_: Particulate Matter ≤ 10 μm; PM _2.5_: Particulate Matter ≤ 2.5 μm; CO: Carbon Monoxide; NO: Nitric Oxide; NO_2_: Nitric Dioxide; SO_2_: Sulfur Dioxide; O_3_: Ozone; NO_x_: Nitric Oxides.

*** ppb: part per billion; ppm: part per million

### Characteristics of the included studies

The included studies were carried out in four countries: 4 studies in the USA, 2 in Taiwan, 1 in Sweden and 1 in Denmark. Seven of the included studies were cohort studies with core aim of investigating the association between AAP and GDM (43–49). And, one study was case-control seeking to assess the effect of developing GDM among women giving preterm birth. The sample size ranged from 2093 to 410267. GDM cases in the included studies were 35605. Six studies used residential-level exposure measurement to estimate the level of the pollutants (42–46, 48). Women exposure were linked to her hospital referral region. Moreover, air pollutants levels were estimated from some distributed air quality monitoring stations.

### Carbon monoxide (CO) (per 1 ppm) and GDM

**Fig. 2:**
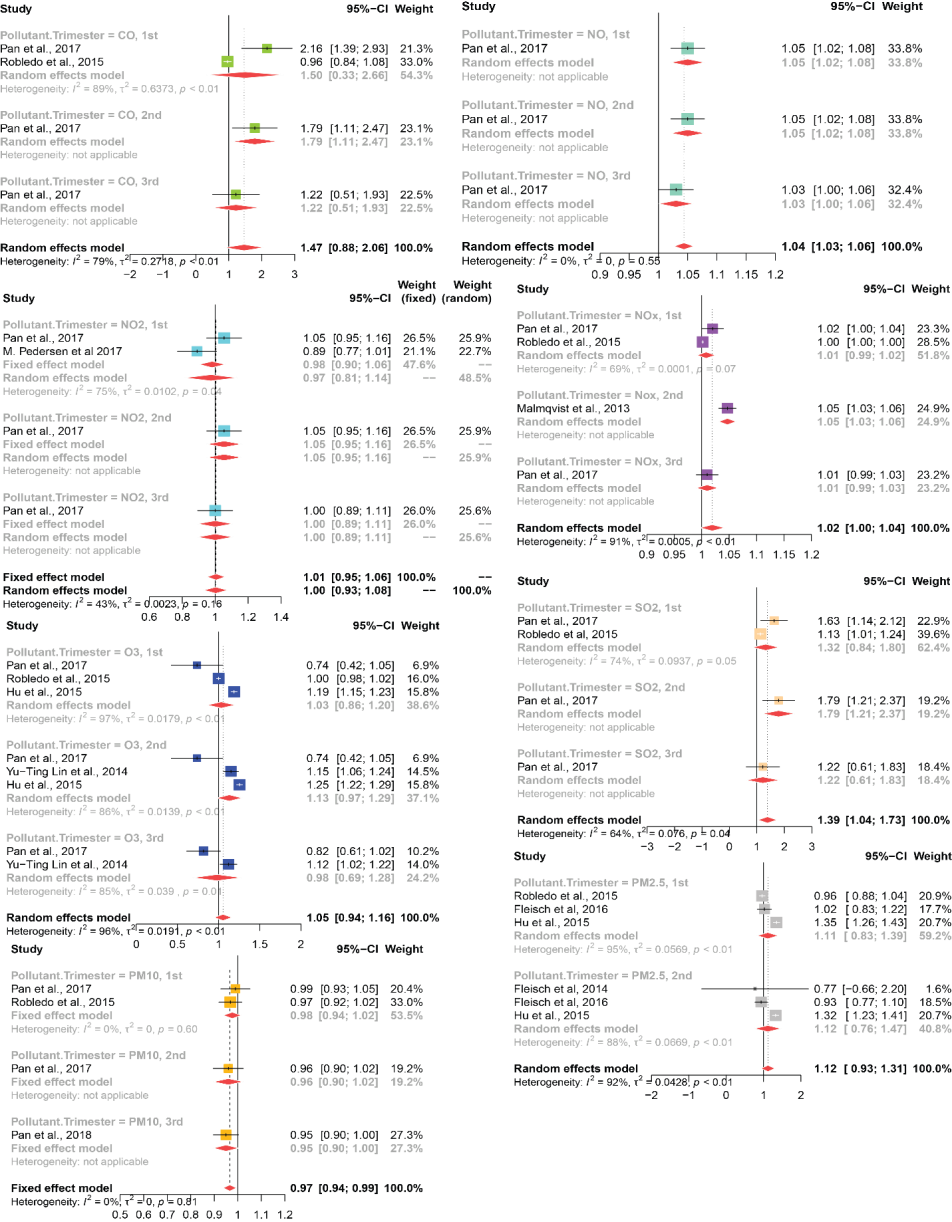
Forest plot of the association between main outdoor air pollutants and GDM

Only two studies investigated the association between GDM and CO (42, 47). No higher risk of developing GDM was showed during the 1^st^ trimester. However, the association between GDM and CO were analyzed during the 1^st^, 2^nd^ and 3^rd^ trimesters and showed higher odds of GDM during them (Fig. 2). The pooled EE was 1.47 (95% CI 0.88–2.06). However, there is a high degree of heterogeneity with I-square of 79%. This could be assigned to the difference in the population, sample size, data sources and exposure measurement methods. Data were utilized from a consortium cohort study including 19 hospitals in the USA linking women exposure to her hospital referral region. Whereas data were used from a national birth registration database in Taiwan measuring air pollutants levels at the residential level. In addition, the sample size number of GDM cases were much more in one study than in other. Moreover, the Taiwanese study accounted for many covariates not adjusted in the USA study including pre-pregnancy body mass index (BMI), weight gain, parity, education and household income (42, 47).

### Nitric oxide (NO) (per 1 ppb) and GDM

Only one study investigated the association between NO and GDM. It found significantly high odds of GDM in all trimesters (Fig. 2). The pooled EE was 1.04 (95% CI 1.03–1.06).

### Nitric dioxide (NO_2_) (per 10 μg/m^3^) and GDM

Two studies assessed the association between NO_2_ and GDM (Fig. 2) (42, 43). Effect estimate of NO_2_ were reported during the first trimester only. The overall effect estimate has low degree of heterogeneity (I-square = 43%, *P*=0.16). The pooled EE was 1.01 (95% CI: 0.95–1.06). Despite that both studies included large sample sizes, used residential-level air monitoring and adjusted for many covariates, there is inconsistency in their 1^st^ trimester results. This could be explained in light of using different GDM diagnostic criteria. A study using the American Diabetes Association criteria; whereas other study used the Danish criteria. Moreover, populations’ difference may be due.

### Nitric oxides (NO_x_) (per 1ppb) and GDM

Three studies reported the association between NO_x_ and GDM (42, 46–47). All studies showed a slightly positive association between NO_x_ and the development of GDM (Fig. 2). The pooled EE was 1.02 (95% CI 1–1.04), I-squared = 91%. In an attempt to clarify the cause of this substantial heterogeneity, each study was removed at a time. When either of the first two studies were removed, substantial heterogeneity still existed (42, 47). However, when the third study was removed, heterogeneity resolved (*P*=0.15) (46). In each case, the odds of GDM was marginally positive. The difference could be discussed in terms of geographical regions differences and study design. Study (46) was undertaken in Sweden, where air pollutants levels were generally below the WHO air quality guidelines levels. Therefore, the effect of lifetime exposure could attribute the difference. In contrast to third study (46), the first two studies (42, 47) studies investigate more than one pollutant in their studies.

### Ozone (O_3_) (per 10 ppb) and GDM

Four studies included the relationship between O_3_ and GDM. All studies, but study (42) showed either no or a positive association between O_3_ and GDM (Fig. 2). The pooled EE was 1.05 (95% CI 0.94–1.16). However, heterogeneity was substantial with I-squared=96%. To investigate it, each study was removed at a time. Heterogeneity remained high each time, except when removing study (45) (I-squared=78%). Despite testing whether the difference in the exposure measurement method, country or by adjusting for season of conception could underlie this, heterogeneity remained substantial. The reason behind the decrease in I-squared when Hu et al., study was removed could be attributed to its sample inclusion and exclusion criteria. Hu et al., applied various exclusion criteria including excluding women whose address could not be geo-coded, preterm birth and births with weight < 400.

### Sulfur dioxide (SO_2_) (per 10 ppb) and GDM

Two studies measured and investigated the association between SO_2_ and GDM (42, 47). Both studies showed high odds of GDM in relation to SO_2_ (Fig. 2). The pooled EE was 1.39 (95% CI 1.04–1.73), with I-squared = 64%). The moderate degree of heterogeneity could be explained by the difference in population, region, data sources and adjustment for covariates. In contrast to study (47), study (42) adjusted its model for BMI, weight gain, and parity.

### Particulate Matter ≤ 10 μm (PM_10_) (per 10 μg/m^3^) and GDM

Two studies reported no association between PM_10_ and the development of GDM (Fig. 2) (42, 47). The pooled EE was 0.97 (95% CI 0.94–0.99), with I-squared = 0%.

### Particulate Matter ≤ 2.5 μm (PM_2.5_) (per 10 μg/m^3^) and GDM

Four studies assessed the effect of PM_2.5_ on developing GDM (Table 1) (44–45, 47–48). The pooled EE was 1.12 (95% CI 0.93–1.31), with I-squared=92% (Fig. 2). When each study was removed at a time, heterogeneity remained, except when removing Hu et al., study. Upon removing Hu et al., study the effect estimate showed no association with pooled EE of 0.96 (95% CI 0.90–0.99), with I-squared=0%. This decrease in the heterogeneity could be explained by the exclusion filters that the Hu et al., applied other than the other studies. For example, it excluded women with birth < 400.

### Quality assessment

Using the modified version of the NOS for quality assessment, four studies got a score of four (42, 43, 44, 46), two studies got a score of three (45, 48), one study scored two (47) and one study got a score of one (49). Thus, six of the included studies were of good quality, whereas two studies were of poor quality.

## Discussion

This systematic review and meta-analysis included a summary of the current evidence regarding the association between ambient air pollution (AAP) and the development of gestational diabetes mellitus (GDM). The effect estimates of the relationship between GDM and air pollutants ranged from 0.97 (95% CI 0.94–0.99) for PM_10_ to 1.47 (95% CI 0.88–2.06) for CO. However, only NO and SO_2_ showed statistically significant effect estimates. In most studies, the second trimester was the most vulnerable period.

Although the biological mechanisms by which AAP contributes to the development of insulin resistance (IR) and glucose intolerance remain unclear, recent experimental and epidemiological have uncovered many insights into the role of AAP-mediated insulin resistance (50). The role of environmental pollutants was evident from the consistent report of the role of persistent organic phosphate pollutants in the development of IR (51–55). In addition, exposure to AAP has been reported to be associated with endothelial dys-function elevated levels of inflammatory mediators including tumor necrosis factor (TNF) α, prostaglandin (PG) E2, C-reactive protein, inter-leukin-1β, and endothelin-1 (56–58). The increased level of inflammation mediator has been shown to interrupt and inhibit insulin signaling and transaction (59–61). Moreover, experimental studies link AAP exposure and endoplasmic reticulum (ER) stress-induced apoptosis in the lung and liver tissue along with brown adipose tissue dysfunction (62–64). ER stress enables the unfolded protein response (UPR) which contributes to the development of IR via inflammation, lipid accumulation, insulin biosynthesis and β-cell apoptosis (65–68). Thus, the accumulative experimental and epidemiological evidence strongly suggest a biological association between AAP and IR.

### Limitations

Some limitations should be taken into consideration when interpreting the results of this review. First, the number of available studies was limited which may lead to restricting the ability to get a more precise estimate with minimal heterogeneity. Second, two of the included studies did not use residential-level pollutant estimation. Thus, this may have led to misclassification of the exact estimate. Finally, most studies have not accounted for many potential covariates and have not applied multipollutant models. Thus, the results of this meta-analysis should be interpreted carefully.

## Conclusion

Some ambient air pollutants may contribute to the development of gestational diabetes mellitus. Reflecting the increasing prevalence of GDM in the context of T2D and obesity necessitates promoting awareness among pregnant women about how air pollution could affect their health and their newborns, especially in regions with limited health care. Besides, this review provides additional evidence about the importance of implementing government-level actions to improve air quality.

## Recommendations

More studies using large, representative sample size and residential-level air monitoring are needed. In addition, they need to take into consideration potential covariates including occupational exposure, indoor air pollution, BMI, ethnicity/race, physical activity, socio-economic background, diet, mother mobility during pregnancy, previous family history of diabetes, and effects of multipollutant rather than one pollutant.

## Ethical consideration

Ethical issues (Including plagiarism, informed consent, misconduct, data fabrication and/or falsification, double publication and/or submission, redundancy, etc.) have been completely observed by the author.
